# Glycosaminoglycan Binding and Non-Endocytic Membrane Translocation of Cell-Permeable Octaarginine Monitored by Real-Time In-Cell NMR Spectroscopy

**DOI:** 10.3390/ph10020042

**Published:** 2017-04-15

**Authors:** Yuki Takechi-Haraya, Kenzo Aki, Yumi Tohyama, Yuichi Harano, Toru Kawakami, Hiroyuki Saito, Emiko Okamura

**Affiliations:** 1 Faculty of Pharmaceutical Sciences, Himeji Dokkyo University, 7-2-1 Kamiohno, Himeji 670-8524, Japan; haraya@nihs.go.jp (Y.T.-H.); aki@gm.himeji-du.ac.jp (K.A.); ytohyama@himeji-du.ac.jp (Y.T.); harano@gm.himeji-du.ac.jp (Y.H.); 2 Institute for Protein Research, Osaka University, 3-2 Yamadaoka, Suita, Osaka 565-0871, Japan; kawa@protein.osaka-u.ac.jp; 3 Department of Biophysical Chemistry, Kyoto Pharmaceutical University, 5 Nakauchi-cho, Misasagi, Yamashina-ku, Kyoto 607-8414, Japan; hsaito@mb.kyoto-phu.ac.jp

**Keywords:** glycosaminoglycan, heparin, cell penetrating peptide, octaarginine, non-endocytic membrane translocation, in-cell nuclear magnetic resonance spectroscopy

## Abstract

Glycosaminoglycans (GAGs), which are covalently-linked membrane proteins at the cell surface have recently been suggested to involve in not only endocytic cellular uptake but also non-endocytic direct cell membrane translocation of arginine-rich cell-penetrating peptides (CPPs). However, in-situ comprehensive observation and the quantitative analysis of the direct membrane translocation processes are challenging, and the mechanism therefore remains still unresolved. In this work, real-time in-cell NMR spectroscopy was applied to investigate the direct membrane translocation of octaarginine (R8) into living cells. By introducing 4-trifluoromethyl-l-phenylalanine to the N terminus of R8, the non-endocytic membrane translocation of ^19^F-labeled R8 (^19^F-R8) into a human myeloid leukemia cell line was observed at 4 °C with a time resolution in the order of minutes. ^19^F NMR successfully detected real-time R8 translocation: the binding to anionic GAGs at the cell surface, followed by the penetration into the cell membrane, and the entry into cytosol across the membrane. The NMR concentration analysis enabled quantification of how much of R8 was staying in the respective translocation processes with time in situ. Taken together, our in-cell NMR results provide the physicochemical rationale for spontaneous penetration of CPPs in cell membranes.

## 1. Introduction

Drug delivery using cell-penetrating peptides (CPPs) is one of the most powerful strategies to resolve the poor cell membrane permeability of new bioactive molecules such as oligonucleotides, plasmids, peptides and proteins for therapeutic pharmaceuticals [1]. Arginine- or lysine-rich CPPs can deliver such cargoes into cells in vitro and in vivo [2,3,4]. Although the endocytic pathway has been thought to be significant [5], more than 90% of the delivered cargo become biologically inactive because of lysosomal degradation [6]. CPPs also traverse cell membrane via the non-endocytic pathway at high concentrations, > ~5–10 μM [7,8]. This process is often named as direct membrane translocation or transduction. The mechanism is essentially a physicochemical, energy-independent process in which no receptors are required [9]. Although the direct membrane translocation is an alternative to endocytosis in order to avoid the lysosomal degradation, how cationic CPPs traverse hydrophobic cell membranes is still controversial [10]. 

As a first step of membrane translocation, cationic CPPs are thought to interact with negatively charged, sulfated glycosaminoglycans (GAGs) such as heparan sulfate and chondroitin sulfate which are covalently linked to membrane proteins at the cell surface [11,12,13,14]. The GAG clustering is induced via the electrostatic interaction with CPP, followed by the actin rearrangement that leads to endocytosis [15,16]. On the other hand, the GAG clustering also triggers the direct membrane translocation of CPPs at high CPP concentrations (> 5 μM) [17]. Although CPPs bind to and translocate into GAG-deficient cells and enzymatically GAG-removed cells [12,13,18,19], we have recently reported that the efficiency of the direct membrane translocation of arginine-rich CPPs is correlated with the favorable enthalpy of binding to heparin, of which the binding could be derived from formation of multidentate hydrogen bonding of the arginine residue with sulfate group of heparin [20]. In addition, the previous study has demonstrated that the direct membrane translocation of arginine-rich peptides including octaarginine (R8) is markedly reduced by the chlorate treatment, which prevents sulfation of both heparan sulfate and chondroitin sulfate chains [20]. Based on these facts, the non-endocytic membrane translocation of arginine-rich CPPs would follow three distinct steps: (1) binding to sulfated GAGs at the cell surface; (2) translocation into cells over potential barrier of the hydrophobic cell membrane; and (3) diffusion through the cytosol. However, in situ comprehensive observation and quantitative analysis of the non-endocytic membrane translocation processes are challenging, and the mechanism therefore remains still unsolved [10]. 

So far, almost all membrane translocation studies have relied upon the fluorescent labeling of CPPs or delivered cargo. Despite the high sensitivity, fluorophores are likely to strengthen the interaction of CPPs with lipid membrane [16,21], induce photodamage of lipid bilayer membranes [22], facilitate the uptake into the cell [23], modify the cellular distribution of the CPP [24,25], and change the structural flexibility and conformation of CPP [26]. Recently, an innovative MALDI TOF-MS quantification was reported by using biotin–avidin interaction [27,28,29]. Although the biotinylated CPP at as low as a femtomole scale has been quantified after the incubation with cells, the method has not been able to catch the real-time processes of CPP’s translocation into cells. Recently- developed real-time NMR spectroscopy [30] is a potential technique for the observation of biologically-relevant functions in a natural manner.

In this work, the real-time solution NMR method is applied to natural living cells to investigate the mechanism for non-endocytic membrane translocation of cell-permeable octaarginine (R8). By introducing 4-trifluoromethyl-l-phenylalanine (4CF_3_-Phe) to the N terminus of R8, the direct membrane translocation of ^19^F-labeled R8 (^19^F-R8) into a human myeloid leukemia cell line (HL60) is observed by ^19^F NMR with a time resolution at a minute scale. ^19^F NMR is advantageous because it is sensitive and no background is present in the cell. The small size, large chemical shift range, and 100% natural isotope abundance of the ^19^F nucleus have made the use of ^19^F-labeled peptides and proteins an attractive method for biologically-relevant NMR studies [31,32]. Labeling of R8 with 4CF_3_-Phe is found to be an effective method to detect peptide uptake to cells with minimal perturbation [23]. In addition, ^19^F NMR spectroscopy enables us to make a quantitative (concentration) analysis relevant to the molecular dynamics of biological interest without perturbing the system [33,34,35,36]. Here we observe the direct membrane translocation of ^19^F-R8 at 4 °C, the temperature low enough to assure no endocytic pathway of the cellular uptake [37]. The method can detect the successive processes of ^19^F-R8 translocation: (1) ^19^F-R8 binds to GAG at the cell surface; (2) penetrates into the cell membrane; and (3) finally enters the cytosol through the membrane. In addition, ^19^F NMR concentration analysis quantifies how much of ^19^F-R8 is in the processes (1)–(3) with time. The information is valuable because the analysis of time-resolved drug transport has been limited to the uptake of a small drug-like ion via the *Escherichia coli* membrane by using second harmonic generation [38]. We also confirm the ^19^F-R8 uptake to the cytosol of HL60 cells using cell fractionation after equilibrium was attained in the real-time NMR measurement. Finally, the most plausible mechanism of the non-endocytic ^19^F-R8 entry into the cell is discussed.

## 2. Results

### 2.1. Real-Time In-Cell ^19^F NMR Spectra

**Figure 1:**
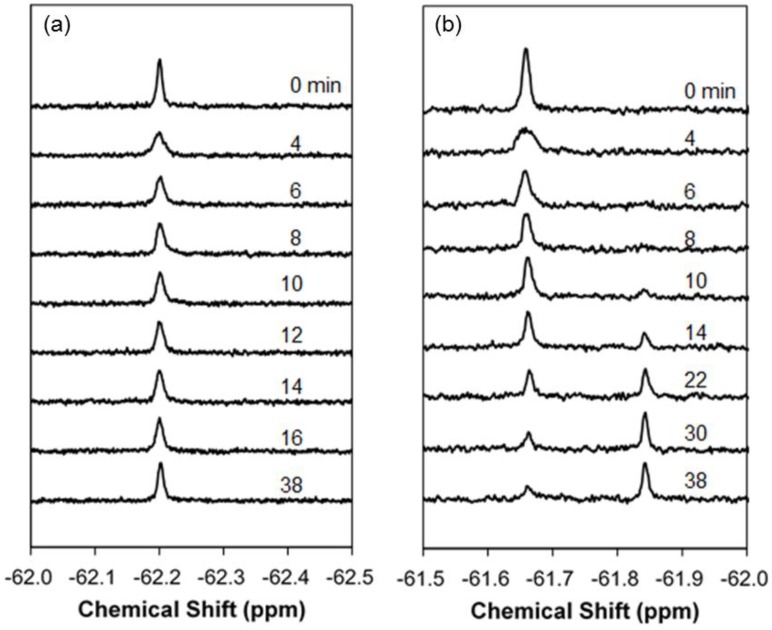
Real-time ^19^F NMR spectra of ^19^F-labeled R8 (^19^F-R8) after addition to HL60 cells at (**a**) 4 and (**b**) 37 °C. The number attached to each spectrum indicates the passage of time before (0 min) and after the addition to cells (in min unit). The ^19^F-R8 concentrations and pH values are (**a**) 80 μM at pH 7.4 and (**b**) 150 μM at pH 7.3, respectively. At 37 °C (**b**), a new peak is observed at −61.84 ppm after 10 min, and gradually increased with time. The increase of the peak at −61.84 ppm is coupled with a gradual decrease in the original signal of ^19^F-R8 at −61.66 ppm. Notice that such kind of signal change is not observed at 4 °C (**a**).

To capture the real-time process of non-endocytic membrane translocation of ^19^F-R8, the solution ^19^F NMR measurement was performed at 4 °C with a time resolution at a minute scale. In order to confirm no contribution of endocytosis at 4 °C, the comparative measurement was also performed at 37 °C. Figure 1a,b shows the real-time ^19^F NMR spectra of ^19^F-R8 before (0 min) and after the addition to HL60 cells at 4 and 37 °C, respectively. At 4 °C (Figure 1a), a signal is observed at −62.20 ppm, that is assignable to the F nuclei of 4CF_3_-Phe at the N terminus of R8. The assignment is confirmed by Figure S1 where signals of 4CF_3_-Phe at −62 ppm and trifluoroacetate (TFA) counter anions at −76 ppm are present with an intensity ratio (4CF_3_-Phe/TFA) of 1:8. At 37 °C, the ^19^F-R8 signal was shifted to −61.66 ppm (Figure 1b). In addition, a new peak was observed at −61.84 ppm after 10 min, and gradually increased with time. The increase of the peak at −61.84 ppm was coupled with a gradual decrease in the original signal at −61.66 ppm. The appearance of a new peak with the disappearance of the original one is due to the presence of cells because such kind of signal changes is not observed in the absence of cells at 37 °C (spectra not shown). Thus the new peak observed at 37 °C is thought to be the result of endocytosis involving peptide degradation [39]. Since such kind of spectral change is not found in Figure 1a, it is reasonable to consider that no endocytosis occurs at 4 °C. The absence of endocytosis at 4 °C is also consistent with the previous results of cell-penetrating peptides [8,9].

**Figure 2:**
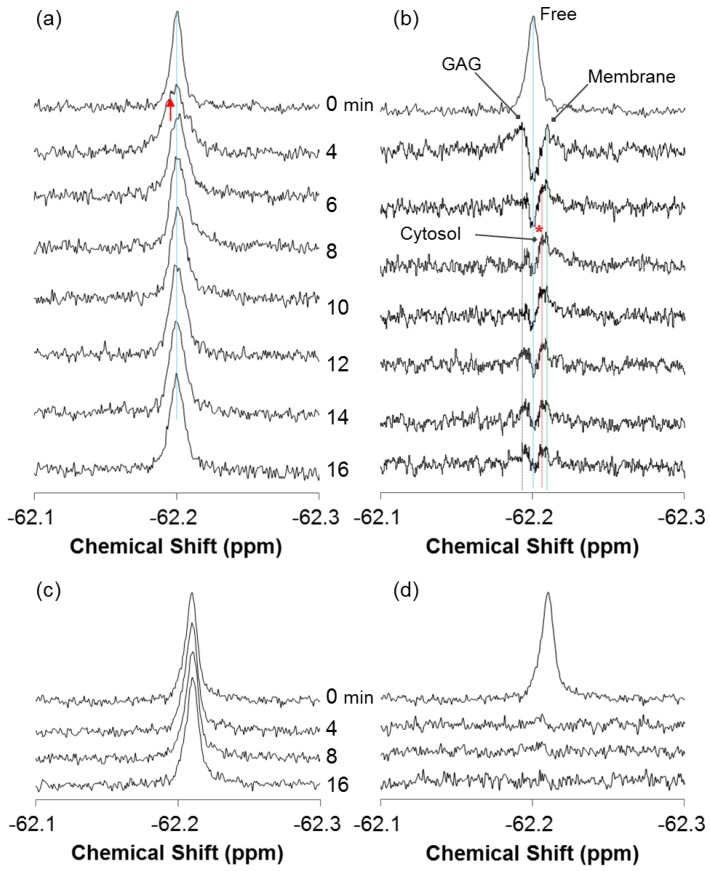
Real-time in-cell ^19^F NMR spectra of ^19^F-R8 and ^19^F-T6 at 4 °C. (**a**) An expansion of the typical ^19^F NMR spectra of 80 μM ^19^F-R8 (Figure 1a) in PBS (0 min), and 4, 6, 8, 10, 12, 14 and 16 min after the addition to HL60 cells at 4 °C. The peak top of ^19^F-R8 in the absence of cells (0 min) is designated by blue dotted line. The red arrow indicates a new component observed at the first step after addition to cells. (**b**) The difference spectra obtained by subtracting the spectrum of ^19^F-R8 in PBS (0 min) from the respective spectra of Figure 2a in the presence of HL60 cells. Note that the top spectrum (Free) in Figure 2b is the ^19^F NMR spectrum of ^19^F-R8 in PBS (0 min). Four components of ^19^F-R8 in cell outside (Free), bound to glycosaminoglycan (GAG), bound to cell membrane (Membrane), and in cytosol (Cytosol, *) are designated by the dotted lines in blue, black, green, and red, respectively. For comparison, the real-time in-cell ^19^F NMR (**c**) and the difference spectra (**d**) of 100 μM ^19^F-T6 in PBS (0 min), and at 4, 8, and 16 min after the addition to HL60 cells are also shown. The upper spectrum in (**d**) represents ^19^F-T6 in PBS (0 min).

Figure 2a shows an expansion of the real-time ^19^F NMR spectra of ^19^F-R8 at 4 °C in PBS (0 min) and 4, 6, 8, 10, 12, 14 and 16 min after the addition to HL60 cells. In comparison to the spectrum in PBS (0 min), the signal is broadened due to the appearance of new component (red arrow) at the low magnetic field within the first 4 min after ^19^F-R8 was incubated with cells. We call it state I. After 6 min, the signal comes back to the high field and becomes sharper (state II). This is because the low field component gradually decreases in intensity during the period from 4 to 6 min. After 8 min, however, the peak top of the signal slightly moves to the lower field again (state III). No further change is observed in the ^19^F-R8 signal after 10 min and later, indicating that the system reaches an equilibrium state. 

As mentioned above, the time-dependent spectral changes in Figure 2a imply that at least three different states I-III of ^19^F-R8 are present after the addition to HL60 cells. We repeated in-cell NMR measurement three times, and confirmed such states every time of the measurement. To distinguish states I, II, and III clearly, it is convenient to see the difference spectrum. The difference spectrum analysis is useful in the present study because the integral intensity of ^19^F-R8 is conserved all the time (see Supplementary Materials Figure S2); notice that no degradation of ^19^F-R8 is induced at 4 °C by the presence of HL60 cells. By subtracting the spectrum of ^19^F-R8 in PBS (0 min) from each spectrum after 4, 6, 8, 10, 12, 14 and 16 min with cells, we can obtain the difference spectra as illustrated in Figure 2b.

The time course of the difference spectra shows that probably three components of ^19^F-R8 are present after ^19^F-R8 is added to HL cells, in addition to the free component at −62.20 ppm. At first, two peaks are observed at −62.19 and −62.21 ppm after 4 min. These peaks can be assigned to ^19^F-R8 bound to GAG (GAG in Figure 2b) and ^19^F-R8 that interacts with the cell membrane (Membrane). Details of the assignment will be described later. Then, the third peak appears at −62.205 ppm after 6 min and increases in intensity after 8 min; see asterisk in Figure 2b. This peak can be assigned to ^19^F-R8 in cytosol (Cytosol) after passing through the membrane. 

**Figure 3:**
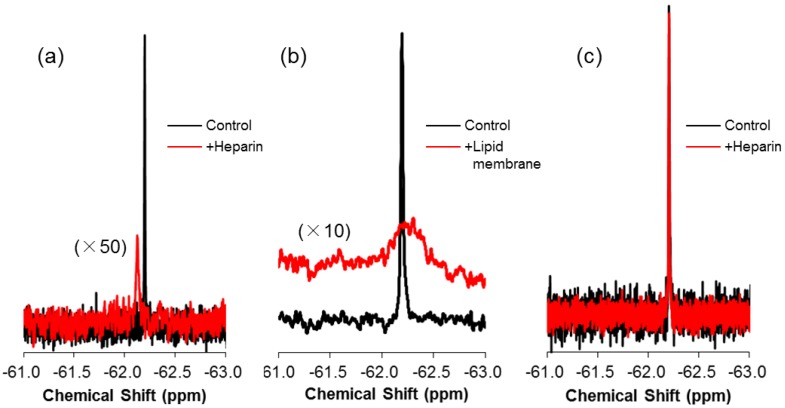
^19^F NMR spectra of ^19^F-R8 in the presence of heparin and lipid membrane. ^19^F NMR spectra of 80 μM ^19^F-R8 in the presence (red) and absence (black) of (**a**) 80 μM heparin and (**b**) a 40-mM EPC/EPG bilayer membrane at 4 °C (pH 7.4). The spectra of 100 μM ^19^F-T6 in the presence of 200 μM of heparin is also shown in (**c**) for comparison.

**Figure 4:**
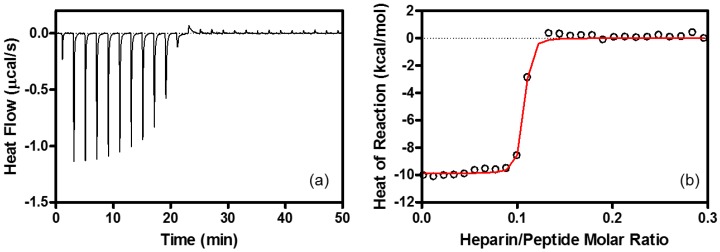
ITC associated with the interaction between R8 and heparin. (**a**) ITC for heparin (100 μM) injection into R8 (45 μM) at 25 °C. Each peak in heat flow chart corresponds to the injection of 1.0 μL aliquots of heparin. (**b**) Heat reactions (integrated from the calorimetric trace, and corrected for the dilution control) plotted as a function of heparin/peptide molar ratio. The solid line is the best fit to the experimental data. Buffer: 10 mM Tris-HCl buffer containing 15 mM NaCl at pH 7.4. The calculated parameters are listed in Table 1.

**Figure 5:**
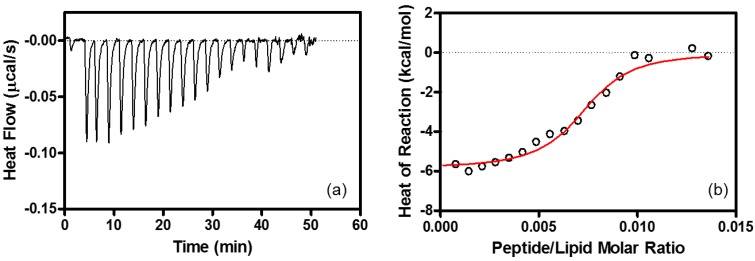
ITC associated with the interaction between R8 and lipid membrane. (**a**) ITC for R8 (51 μM) injection into EPC/EPG LUV (500 μM) at 25 °C. Each peak in heat flow chart corresponds to the injection of 2.0-μL aliquots of R8. (**b**) Heat reaction (integrated from the calorimetric trace, and corrected for the dilution control) plotted as a function of peptide/lipid molar ratio. The solid line is the best fit to the experimental data. Buffer: 10 mM Tris-HCl buffer containing 15 mM NaCl at pH 7.4. The calculated parameters are listed in Table 1.

It is noted that the peak assignments are reasonable in view of the following results of ^19^F NMR and isothermal titration calorimetry (ITC). The first is that the NMR chemical shift of ^19^F-R8 moves toward the low magnetic field as compared to ^19^F-R8 in PBS when ^19^F-R8 is mixed with heparin; see Figure 3a. Because heparin is frequently used as a model of GAG [40,41,42,43,44,45], it is reasonable to assign the broad component at −62.19 ppm to ^19^F-R8 that is bound to GAG. According to the fact that NMR signal intensity is reduced by slower rotational movement of a molecule related to short transverse relaxation time, it should be noted that the rotational dynamics of ^19^F-R8 are restricted due to the tighter binding to heparin, as previously discussed [20]. The high affinity of R8 for heparin is confirmed by the ITC result in Figure 4 that leads the association constant 1.3 × 10^8^ M^−1^, and the binding free energy, −10.9 kcal/mol at 25 °C, as listed in Table 1. The binding nature of R8 is largely derived from the electrostatic interaction between arginine residues and anionic sulfate/carboxyl groups of heparin [20]. The binding stoichiometry (molar ratio of peptide/heparin = ~11) corresponds approximately to the ratio for the charge neutralization (the heparin molecule used possesses an average of 80 anionic charges, whereas there are 8 cationic charges of octaarginine). The assignment also corresponds well with the previous consensus that R8 at first comes contact with GAG at the cell surface by the electrostatic interaction [46]. The second is that the ^19^F NMR signal moves to a high magnetic field where ^19^F-R8 interacts with cell membrane, in contrast to the electrostatic ^19^F-R8 binding to GAG. As illustrated in Figure 3b, it is confirmed that the ^19^F-R8 signal shifts to a high field after the binding to large unilamellar vesicle (LUV) composed of egg phosphatidylcholine (EPC) and egg phosphatidylglycerol (EPG) as model cell membrane. The result is consistent with the observation that the chemical shift of the ^19^F NMR signal moves to the higher magnetic field when ^19^F molecules are in a hydrophobic environment [23,47,48]. Similar to the case of heparin binding, the NMR signal intensity of ^19^F-R8 is also reduced due to the binding to EPC/EPG LUV. The presence of energetically-favorable interaction between R8 and EPC-EPG membrane is also demonstrated by the ITC result in Figure 5 that gives the association constant, 1.5 × 10^6^ M^−1^ and the binding free energy, −8.4 kcal/mol at 25 °C (Table 1). The binding stoichiometry (molar ratio of lipid/peptide = ~100) is close to the ratio for charge neutralization, that is, the molar ratio of EPG in the outer leaflet of the LUVs to the positively charged residues of octaarginine is ~1. Thus we can consider that the most plausible assignment of the peak at −62.21 ppm is ^19^F-R8 in the cell membrane. Finally, it is reasonable to assign the third peak at −62.205 ppm (*) as ^19^F-R8 in cytosol, because the peak comes back to the lower magnetic field due to rather hydrophilic cytosol environment as compared to the cell membrane. The presence of ^19^F-R8 in cytosol is also confirmed by cell fractionation, the details of which will be described later.

Based on the assignment of the ^19^F NMR spectra in Figure 2, here we propose a hypothesis about the most probable mechanism of non-endocytic membrane translocation of ^19^F-R8 to HL60 cells as the following: (1) ^19^F-R8 first binds to GAG (state I); (2) penetrates into cell membrane (state II); and (3) finally enters the cytosol (state III). In Figure 2b, the three components of ^19^F-R8 bound to GAG, ^19^F-R8 in membrane, and in cytosol are demonstrated by the dotted lines in black, green, and red, respectively, together with the free component (Free) in blue. It is found that the signal of ^19^F-R8 bound to GAG and that in membrane already appear 4 min after the addition to cells. The GAG signal quickly decays with time and almost disappears after 6 min. Meanwhile, the membrane signal decays slowly as compared to GAG. Then, the signal in cytosol (Cytosol) is identified at 6 min, a few minutes after GAG and Membrane peaks are observed. The Cytosol signal is gradually increased with time and almost unchanged after 10 min, to confirm the equilibrium state of the translocation of ^19^F-R8 to HL cells. The observed minute-ordered direct membrane translocation of ^19^F-labeled octaarginine is consistent with our previous study that has confirmed the cell penetration of fluorescently-labeled octaarginine within at least 30 min [20]. There have also been reported that the fluorescently-labeled R8 and biotin-labeled nonaarginine penetrate into cells after about 5 min at 4 °C [7,8].

To verify the reliability of the analysis, a membrane-impermeable human lens αA-crystallin fragment, called ^19^F-T6 (TV-(4CF_3_-Phe)-DSGISEVR), was added to HL60 cells, and the spectra were compared. As ^19^F-T6 includes two acidic and one cationic amino acids, the negative net charge is held under physiological conditions. The interaction between ^19^F-T6 and negatively charged GAG is, therefore, not expected at the cell surface. In fact, as shown in Figure 2c,d, no changes were found in the ^19^F NMR spectrum nor the difference spectrum of ^19^F-T6 even 16 min after the addition to cells. The situation is a sharp contrast to ^19^F-R8 where the equilibrium has been already attained for the membrane translocation process. The spectrum of ^19^F-T6 was not changed even after 46 min (data not shown). The result demonstrates that no interaction occurs between ^19^F-T6 and HL60 cells. This is also supported by the fact that the spectra of ^19^F-T6 are not altered after it is added to heparin (Figure 3c), indicating no binding of ^19^F-T6 to negatively charged GAG on the cell surface. 

### 2.2. Quantitative Analysis of Direct Membrane Translocation

**Figure 6:**
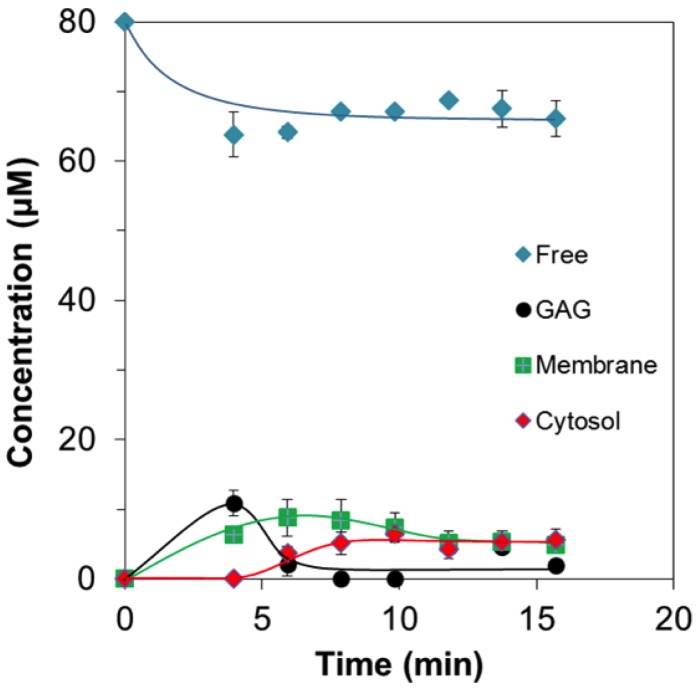
Real-time changes of ^19^F-R8 concentrations in HL60 cells. The ^19^F-R8 concentrations in outside (blue), bound to GAG (black), bound to membrane (green), and in cytosol (red) of HL60 cells at 4 °C are shown as a function of time. Each symbol represents the experimental value from the NMR signal intensity. Solid lines represent a visual guide.

So far, no reports about in-situ quantity of CPPs in cells have been available. Here, by using the integral signal intensities of the real-time ^19^F NMR difference spectra (Figure 2b), the quantities of four ^19^F-R8 components, Free, GAG, Membrane, and Cytosol can be evaluated as a function of time. Detailed procedures are described in Appendix A. Figure 6 quantifies how the concentration of each ^19^F-R8 component varied after the addition to HL60 cells. The amount of free ^19^F-R8 gradually decreased for the first 5 min. This corresponds to the uptake of free ^19^F-R8 to HL60 cells via the binding to GAG. At least 65 μM (81%) of ^19^F-R8 was, however, remaining in a free state after the equilibrium was attained (at 16 min). The amount of ^19^F-R8 bound to GAG, at first, increased but decreased to less than 5 μM within a short period of 4–6 min. This quick decrease is thought to be due to the transfer of ^19^F-R8 to the membrane from the cell surface. In fact, the amount of ^19^F-R8 in the membrane was gradually increased to 9 μM at 6 min, and then slightly decreased. The decrease in ^19^F-R8 in membrane suggests that the peptide was delivered to cytosol after passing through the membrane. Actually, the transfer of ^19^F-R8 to cytosol was first observed at around 5 min, followed by the increase up to about 6 μM.

It should be noted that the movement of ^19^F-R8 from cytosol to the membrane occurs as frequently as the entry into cytosol because the concentrations of ^19^F-R8 in membrane and in cytosol at the equilibrium state after 16 min are found to be equal within the experimental error. The relatively low concentrations are both reasonable from the fact that cell membranes impose a hydrophobic barrier on highly cationic R8 [49,50].

### 2.3. ^19^F-R8 Distribution under Equilibrium

**Scheme 1:**
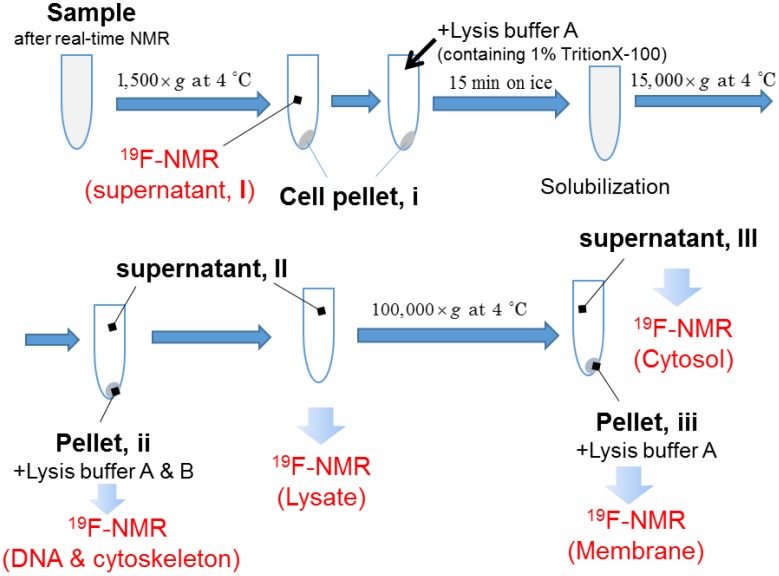
Procedures of cell fractionation after real-time NMR measurement.

**Figure 7:**
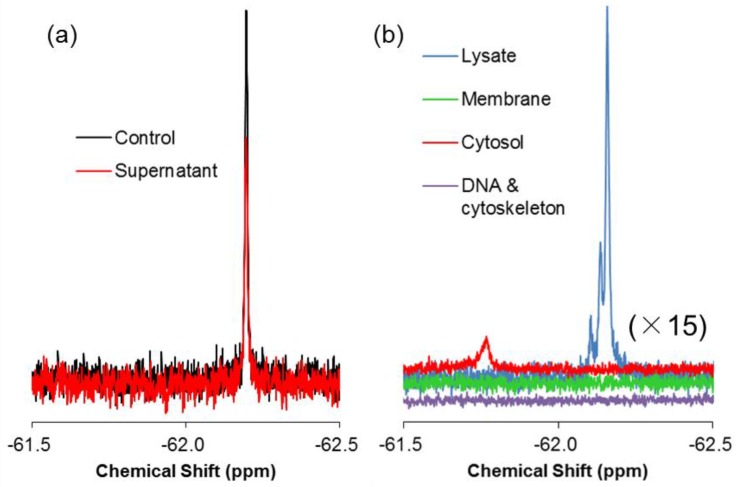
Final distribution of ^19^F-R8 in HL60 cells. (**a**) ^19^F NMR spectra of ^19^F-R8 in PBS (control) and the supernatant **I** after real-time in-cell ^19^F NMR measurement at 4 °C. The spectra were obtained at 4 °C, pH 7.4. (**b**) ^19^F NMR spectra of ^19^F-R8 fractions separated by solubilization and centrifugation in accordance with Scheme 1. All spectra were observed at 4 °C and pH 7.5. The signal of cytosol fraction is shifted as a result of the interaction with concentrated lysis buffer components, because supernatant III, containing lysis buffer A, was lyophilized and dissolved in large amount of lysis buffer again.

In the previous sections, we succeeded in comprehensive observation and quantitative analysis of the non-endocytic translocation of ^19^F-R8 to the cell inside. To confirm that ^19^F-R8 is actually transferred to cytosol across the cell membrane, the final distribution of ^19^F-R8 was evaluated by cell fractionation after equilibrium was attained in real-time NMR measurements. Membrane solubilization and centrifugation techniques were combined in accordance with Scheme 1. First, we examined how much of ^19^F-R8 was finally bound to cells. In Figure 7a, the ^19^F NMR spectra of ^19^F-R8 in the supernatant **I** is compared with total ^19^F-R8 as a control at 4 °C. The ^19^F NMR signal intensity of the supernatant **I** corresponds to ^19^F-R8 that is still in a free (unbound) state under equilibrium. It is found that 77% of ^19^F-R8 was in a free state. The value is consistent with the result of the real-time in-cell NMR measurement showing about 65 μM (81%) of ^19^F-R8 is remaining in a free state after the equilibrium is attained (Figure 6). Next, to confirm that ^19^F-R8 is actually bound to HL60 cell, the cell pellet **I** was solubilized by lysis buffer containing 1% Triton X-100. After the centrifugation, the supernatant **II** was subject to ^19^F NMR measurement at 4 °C. The spectrum is shown in Figure 7b as Lysate, and 13% of the initial ^19^F-R8 was detected. 

It is considered that three components of ^19^F-R8 are contained in the Lysate. They include ^19^F-R8 bound to GAG or cell membrane, and ^19^F-R8 in cytosol. We separated these components as the supernatant **III** and the pellet **III** by centrifuging the Lysate at 100,000× *g*. Supernatant **III** consists of ^19^F-R8 in cytosol, and pellet **III** contains ^19^F-R8 bound to GAG or cell membrane (referred as Membrane); see Scheme 1. The ^19^F NMR spectrum of the supernatant **III** at 4 °C shows that the signal of ^19^F-R8 is observed in the cytosol fraction; see the red line in Figure 7b. The result is valuable because the ^19^F-R8 entry into cytosol through HL60 cell membranes is actually demonstrated. This is a contrast to the absence of TFA peak in the Lysate fraction (Figure S3), indicating that the counter TFA ions of ^19^F-R8 remain outside cells after the real-time in-cell ^19^F NMR measurement. On the other hand, the ^19^F-R8 in the membrane fraction is found to be within the experimental error at an equilibrium state. Although undesirable loss of peptide may be induced by the extensive solubilization and centrifugation for cell fractionation, almost no appearance of the membrane fraction is probably due to the signal broadening, as seen in spectra of ^19^F-R8 bound to GAG or EPC/EPG LUV (Figure 3a,b). It should be noted that the observed chemical shift of NMR signal in this system is too complicated to understand. For example, the cell solubilization exposes ^19^F-R8 to numerous molecules derived from cells. Also, the cytosol fraction was obtained by lyophilization of 50 mL lysis buffer A. This leads to the increased concentrations of components (Triton X-100, Tris-HCl, EDTA, NaCl in lysis buffer A) in the final sample to be measured. The condition of the solvent such as ionic strength and pH affects the chemical shift of ^19^F-R8 NMR signal. Thus the integral NMR intensity is useful for the steady state NMR spectra because it is basically proportional to the nuclear concentration [51].

As ^19^F-R8 is cationic, it is possible that ^19^F-R8 is finally bound to DNA in the nucleus of HL60 cells. We explored whether ^19^F-R8 was bound to DNA or cytoskeleton by solubilizing pellet **II** in accordance with Scheme 1. As shown in Figure 7b, the ^19^F NMR signal of ^19^F-R8 in the DNA and cytoskeleton was not yet detectable. Although further investigation is required, this may be due to the fact that the binding of ^19^F-R8 to cellular component is too tight to be solubilized. In such case, the intensity of the NMR signal is underestimated by the signal broadening, similar to the case of membrane fraction.

## 3. Discussion

**Figure 8:**
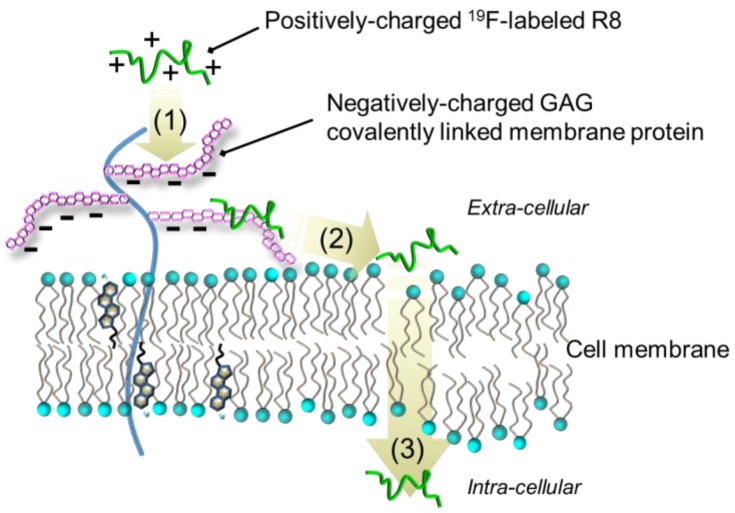
A plausible mechanism for non-endocytic, energy-independent translocation of ^19^F-R8 into cells. The mechanism involves (1) binding of ^19^F-R8 to GAG at the cell surface, followed by (2) the transfer to the cell membrane and (3) the entry into cytosol.

In the present study, we successfully observed the real-time processes of the direct membrane translocation of ^19^F-R8 into HL60 cells without any perturbation of the system. Based on the results, we can discuss the plausible mechanism as illustrated in Figure 8.

As a first step of the entry into cells, cationic ^19^F-R8 electrostatically binds to negatively charged GAGs at the cell surface. Consistent with this, the same conclusion has been reached that arginine-rich CPPs such as R8 at first bind to GAGs at the cell surface [20,46]. Considering the fact that the fraction of acidic charged phospholipids in biological plasma membranes is only about 10%–20%, and that the lipids are predominantly distributed to inner leaflet of the membrane [52], it seems that ^19^F-R8 bound to GAGs remains outside cells. However, contrary to the expectation, quantitative NMR analysis demonstrated the entry of cationic ^19^F-R8 into hydrophobic cell membrane after binding to GAGs (Figure 2b and Figure 6). One possibility is that the charge neutralization of polyarginine with GAGs would lead to insoluble peptide-GAG complexes [14,17,41], which is likely to be energetically unstable since GAGs are covalently immobilized to membrane protein at the water-abundant cell surface [53,54]. As a result, ^19^F-R8 bound to GAG is likely to dissociate from GAGs to water or rapidly transfer to cell membrane. Indeed, it was reported that the cationic ^19^F-R8 bound to the amphipathic sulfate compounds favorably partitions into octanol phase as cell membrane model via hydrophobic interactions [55,56]. In addition, bidentate hydrogen bonding between guanidino group of arginine and lipid phosphate makes the arginine-rich peptides stable in a hydrophobic environment [57,58,59]. Taken together, our results suggest that the charge neutralization of arginine-rich peptides by the presence of negatively charged GAG accelerates the peptide entry into the hydrophobic membrane inside.

Afterwards, ^19^F-R8 is transferred to the cytosol. In order to enter the inside of cells, ^19^F-R8 should pass through the hydrophobic cell membrane. Because ^19^F-R8 is inherently hydrophilic, it is hard to enter the hydrophobic lipid bilayer. 

One possibility to compensate this difficulty is to utilize the lipid movement in the vertical direction to the membrane surface. It is expected that the entry of ^19^F-R8 into the cell is enhanced by synchronization with the vertical fluctuation of the membrane lipid. In fact, such movement, called protrusion, has been observed in the cell-sized lipid bilayer vesicle [60]. In this sense, the lipid protrusion motion is considered as one of the key factors for the direct membrane translocation of CPPs. This is especially the case under physiological conditions at 37 °C. At 4 °C, however, the direct translocation probability of ^19^F-R8 is low due to impaired membrane fluidity. The protrusion is inhibited at low temperatures even in the fluid phase [60]. Recently, it has been reported that the most plausible mechanism for the direct membrane translocation of arginine-rich peptides is a transient pore formation, in which the peptides induce membrane perturbation so that it can easily pass through the membrane [56,61,62,63,64,65]. The lifetime of the toroidal pore is thought to be short enough to guarantee no cytotoxicity [66,67,68]. The trypan blue staining after the NMR measurement showed that ^19^F-R8 did not lower cell viability, being consistent with the transient pore formation model.

In conclusion, the present in-cell NMR study is the first report to comprehensively observe and quantitatively analyze the direct translocation processes of cell penetrating ^19^F-R8 in situ. Based on the results, a new insight into the mechanism for the entry of cationic ^19^F-R8 into hydrophobic membrane after binding to negatively charged GAGs was obtained. The present study shows a potential for elucidating direct membrane translocation mechanism of CPPs with minimal perturbation.

## 4. Materials and Methods

### 4.1. Materials

The ^19^F-labeled octaarginine (^19^F-R8: (4CF_3_-Phe)-RRRRRRRR) was synthesized manually by solid phase synthesis method using Fmoc chemistry. The amino and carboxyl termini of the peptide were acetylated and amidated, respectively. A fragment peptide, called ^19^F-T6 (TV-(4CF_3_-Phe)-DSGISEVR), from human lens αA-crystallin [69] was used as a negative control and synthesized by Fmoc solid-phase chemistry using an automated solid-phase synthesizer (PSSM-8; Shimadzu, Kyoto, Japan). The purity of each peptide was confirmed to be > 95% by reversed-phase high-performance liquid chromatography and MALDI-TOF mass spectrometry. Heparin sodium salt (from porcine intestinal mucosa; average molecular weight, 18,000 Da) was purchased from SIGMA (St. Louis, MO, USA). Egg phosphatidylcholine (EPC, > 96% pure) and egg phosphatidylglycerol (EPG, > 95% pure) were obtained from the NOF CORPORATION (Tokyo, Japan). All other reagents were of special grade and used without further purification.

### 4.2. Cell Culture

A human leukemia cell line HL60 was maintained in RPMI 1640 medium, supplemented with 8% heat-inactivated fetal calf serum (FCS), 100 U/mL penicillin and 100 μg/mL streptomycin in 5% CO_2_ humidified air at 37 °C.

### 4.3. Preparation of Large Unilamellar Vesicle (LUV)

EPC/EPG LUV was prepared by using an extrusion method previously reported [70]. Briefly, EPC and EPG were dissolved in chloroform at a molar ratio of PC/PG = 4/1 in a round-bottomed flask and dried with a rotary evaporator to create a thin and homogeneous lipid film. The lipid film was vortexed in Tris-HCl buffer (pH 7.4) containing 15 mM NaCl to obtain vesicle suspension. The resultant suspension was subjected to five cycles of freeze–thawing and was then passed through a Mini-extruder equipped with two stacked 0.1-μm polycarbonate filters (Avanti Polar Lipids, Alabaster, AL, USA). The concentration of phospholipids was determined by the Bartlett method [71].

### 4.4. Real-Time In-Cell ^19^F NMR Measurement

One-dimensional in-cell ^19^F NMR measurements were carried out at 376.2 MHz by using a JEOL ECA400 NMR spectrometer equipped with a superconducting magnet of 9.4 T. A multinuclear probe (JEOL, NM40T10A/AT) for a 10-mm diameter tube was used. Detailed procedures of the measurement are described elsewhere [23]. Briefly, HL60 cells (the final concentration, 1 × 10^7^ cells/mL) were suspended in phosphate-buffered saline (PBS, pH 7.4) at 4 °C and put into a NMR tube. In order to confirm no contribution of energy-dependent endocytosis at 4 °C, the comparative measurement was also performed at 37 °C. To avoid cellular toxicity, the amount of D_2_O used for the signal lock was decreased to 10%. The sample was gently rotated to prevent the sedimentation of the cells. Field-gradient shimming was applied before the addition of the peptide, to quickly attain the spectral resolution. The measurements started immediately after the thermal equilibrium was attained, 1.5 min after the addition of the ^19^F-labeled peptides. The final concentrations of ^19^F-R8 and ^19^F-T6 were 80 μM and 100 μM, respectively, and these were high enough to observe non-endocytic translocation [7,10,26]. Free induction decays (FIDs) were accumulated at 16 time/2-min intervals. For ^19^F-R8, the in-cell ^19^F NMR measurement was repeated three times. The spectra were processed by the JEOL DELTA software. Chemical shift of the ^19^F NMR signal was obtained by referring to the absorption frequency of the trifluoroacetic acid in the solvent. Cell viability, assessed by the trypan blue staining after the NMR measurement at 4 °C, was 92% ± 1% for ^19^F-R8 and 94% ± 1% for ^19^F-T6 with respect to the control value, 94% ± 2%. At 37 °C, the viability was 92% ± 2% and 95% ± 3% in the presence and absence of ^19^F-R8, respectively. 

### 4.5. Steady State ^19^F NMR Measurement 

The amount of ^19^F-R8 finally delivered to the cytosol was quantified by ^19^F NMR under equilibrium in combination with the cell fractionation using membrane solubilization and centrifugation. The cell fractionation procedures are summarized in Scheme 1. After the real-time ^19^F NMR measurement, 4 mL of the sample were centrifuged at 1500× *g* for 5 min at 4 °C. Pellet **I** was washed twice with 4 mL of PBS and centrifuged again. Then the 8 mL of supernatant **I** was collected and subject to ^19^F NMR measurement at 4 °C, to quantify free ^19^F-R8. Next, 4 mL of lysis buffer A (1% Triton X-100, 50 mM Tris-HCl, 50 mM EDTA, 150 mM NaCl, 10% D_2_O) was added to pellet **I**, and left for 15 min on ice to complete the cell membrane solubilization. The solution was centrifuged at 15,000× *g* for 15 min at 4 °C, to separate pellet **II** and supernatant **II**. Then, supernatant **II** was collected and subjected to ^19^F NMR measurement at 4 °C (called Lysate). After measurement, 50 mL of lysis buffer A was added and centrifuged at 100,000× *g* for 3 h at 4 °C. Pellet **III** contains cell membrane and supernatant **III** corresponded to the cytosol fraction [72]. Pellet **III** was resuspended in 4 mL of lysis buffer A and subjected to ^19^F NMR measurement at 4 °C (called Membrane). On the other hand, supernatant **III** was lyophilized and resuspended in 4 mL of lysis buffer A, and subjected to ^19^F NMR measurement at 4 °C (Cytosol). Pellet **II** was incubated in 1 mL of lysis buffer A containing 0.5 M NaCl for 15 min on ice, and added to 3 mL of lysis buffer B (0.05% SDS, 0.5% deoxycholic acid, 50 mM Tris-HCl, 5 mM EDTA, 150 mM NaCl, 10% D_2_O, pH 7.5). Then the solubilized fractions were subjected to ^19^F NMR measurement at 4 °C (DNA and cytoskeleton). In each experiment, the FIDs were accumulated 10,000–60,000 times to attain a high signal to noise ratio.

### 4.6. Isothermal Titration Calorimetry (ITC)

ITC measurements were carried out on an iTC200 system (MicroCal) at 25 °C in 10 mM Tris-HCl buffer at pH 7.4. Peptide solution was placed in the reaction cell, and titrated with aliquots of heparin, or EPC/EPG LUV. The ITC injections were repeated automatically at 25 °C under 1000 rpm stirring. The heats of reaction were corrected for dilution control. Thermodynamic parameters were determined by non-linear least-square fitting of the data using the single site binding model in program Origin for ITC version 7 (MicroCal) with the stoichiometry (*n*), the enthalpy of the reaction (Δ*H*°), and the association constant (*K*) [45,73,74]. The Gibbs free energy Δ*G*° and entropy Δ*S*° for binding of R8 to heparin or EPC/EPG LUV were obtained by the following equations:

Δ*G*° = −*RT*ln*K* (1)

and

*T*Δ*S*° = Δ*H*° − Δ*G*° (2)

where *T* is the absolute temperature.

## Figures and Tables

**Table 1:** Thermodynamic parameters for the interaction of R8 with heparin or egg phosphatidylcholine (EPC)/egg phosphatidylglycerol (EPG) large unilamellar vesicles (LUVs) obtained from isothermal titration calorimetry (ITC).

	Binding Stoichiometry (*n*)	*K* (M^−1^)	Δ*G°* (kcal/mol)	Δ*H°* (kcal/mol)	*T*Δ*S°* (kcal/mol)
Heparin	R8/heparin = 11 ± 1.1	(1.3 ± 0.22) × 10^8^	−10.9 ± 0.10	−9.6 ± 0.53	1.3 ± 0.54
EPC/EPG LUV	lipid/R8 = 100 ± 10	(1.5 ± 0.25) × 10^6^	−8.4 ± 0.10	−6.3 ± 0.10	2.1 ± 0.10

For original ITC data, see Figure 4 and Figure 5.

**Figure A1:**
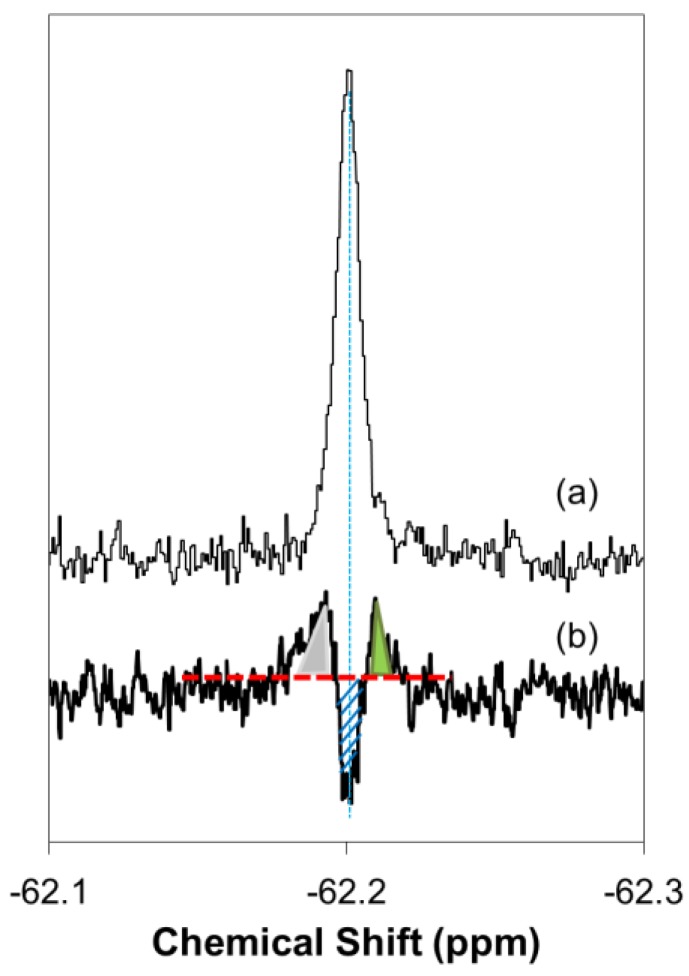
Illustration of the signal intensity estimation in the ^19^F NMR difference spectrum. The spectrum (**a**) shows an ^19^F-R8 signal in PBS (control), and (**b**) is an example of an NMR difference spectrum obtained by subtracting (**a**) from the ^19^F-R8 spectrum 4 min after the peptide was added to cells. The baseline in the difference spectrum is designated by a broken red line. In (**b**), one negative peak at −62.20 ppm and two positive peaks at −62.19 and −62.21 ppm are found. The negative signal (in blue, shaded) is assigned to ^19^F-R8 in a free (unbound) state, and positive ones correspond to ^19^F-R8 bound to GAG (in gray) and cell membrane (in green). The negative peak at −62.20 ppm means the decrease in free peptide. The concentration of these three components can be evaluated from the signal intensities by integrating the respective peak areas (see text).

## Supplementary Materials

The following are available online at http://www.mdpi.com/1424-8247/10/2/42/s1, Figure S1: ^19^F NMR spectrum of ^19^F-R8 solution, Figure S2: Time course of ^19^F NMR signal intensity of ^19^F-R8 after addition to HL60 cells, Figure S3: Final distribution of trifluoroacetate counterions of ^19^F-R8.

## Appendix A

### Concentration Analysis

Cell peptide concentrations inside and outside were estimated by using the signal intensities of real-time ^19^F NMR difference spectra in Figure 2b. Figure A1 illustrates how peptide concentrations were determined from the ^19^F NMR spectra. As compared to the control signal at −62.20 ppm (a), one negative and two positive peaks are found in this case, with respect to the baseline in the difference spectrum (b). The negative peak corresponds to the decreased fraction of free (unbound) peptide because the peak minimum at −62.20 ppm is similar to the control (a). The positive peaks at −62.19 and −62.21 ppm can be assigned to ^19^F-R8 bound to GAG and cell membrane; see Results for peak assignment.

The concentration of these three components can be evaluated from the signal intensities by integrating the respective peak areas. For example, the fraction of the decrease in free ^19^F-R8 component, shaded blue area in the spectrum (b), is estimated as 16.3% with respect to the control, 100% (a). Thus, the free component remaining in cell outside is calculated as 83.7%. Similarly, the increase in the fraction bound to GAG (in gray) and membrane (in green) is estimated as 11.4 and 6.6%. Since the total ^19^F-R8 concentration is 80 μM, the concentrations of free, bound to GAG, and membrane components are calculated to be 67, 9, and 5 μM, respectively.
