# Supplementary material for: Glycosaminoglycan Binding and Non-Endocytic Membrane Translocation of Cell-Permeable Octaarginine Monitored by Real-Time In-Cell NMR Spectroscopy
Source: Pharmaceuticals (Basel). 2017 Apr 15;10(2):42. doi: 10.3390/ph10020042 (PMC5490399; doi:10.3390/ph10020042)
Supplement: Supplementary file 1 [file pharmaceuticals-10-00042-s001.pdf]

Supplementary Materials: Glycosaminoglycan Binding and Non-endocytic Membrane Translocation of Cell-permeable Octaarginine Monitored by Real Time In-cell NMR Spectroscopy

Yuki Takechi-Haraya, Kenzo Aki, Yumi Tohyama, Yuichi Harano, Toru Kawakami, Hiroyuki Saito and Emiko Okamura

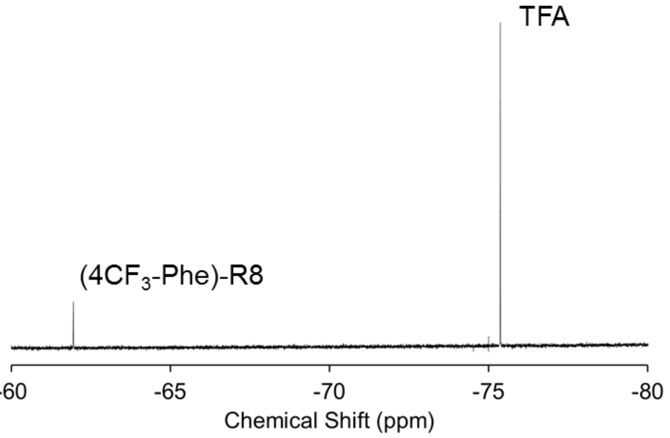

Figure S1. ^{19}F NMR spectrum of ^{19}F-R8 solution in this study. Two peaks are assigned to (4CF_3-Phe)-R8 at -62 ppm and trifluoroacetate (TFA) counteranions at -76 ppm. The assignment is confirmed by the integral intensity of 0.44 (TFA) at -76 ppm with respect to 0.054 (4CF_3-Phe)-R8 at -62 ppm, indicating that the peptide contains 8 TFA counteranions. The signal assignment of ^{19}F-R8 at around -62 ppm is consistent with the previous ^{19}F-NMR study of (4CF_3-Phe)-labeled proteins.[1] It has also been reported that the chemical shift of TFA signal is around -76 ppm.[2]

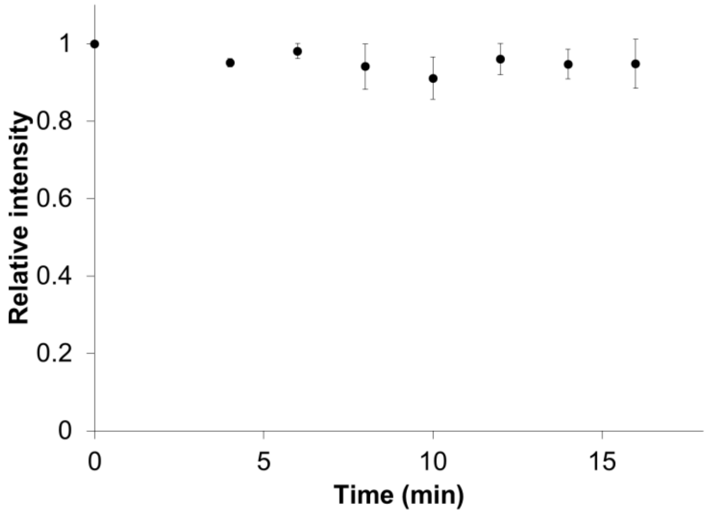

Figure S2. Time course of ^{19}F NMR signal intensity of ^{19}F-R8 after addition to HL60 cells. The intensity is the value relative to the ^{19}F NMR signal of the initial ^{19}F-R8 in PBS. Notice that the ^{19}F-R8 signal intensity is conserved all the time, meaning no degradation of ^{19}F-R8 by the presence of HL60 cells.

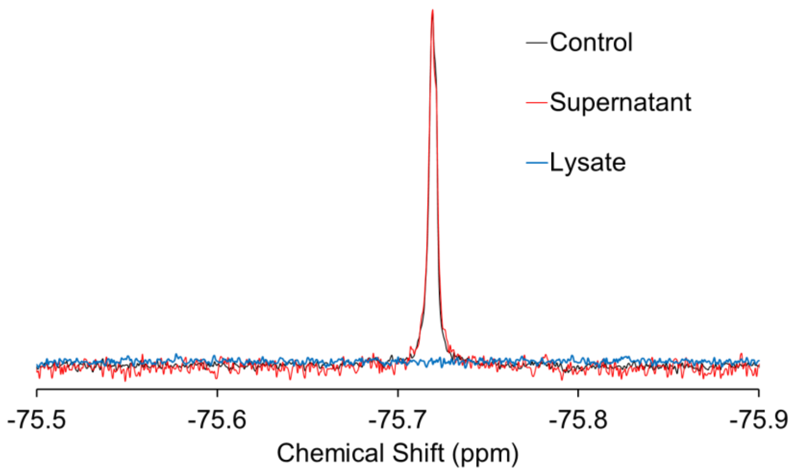

Figure S3. Final distribution of trifluoroacetate counterions of ^{19}F-R8. ^{19}F NMR spectra of trifluoroacetate counterions of ^{19}F-R8 in PBS (control) and in supernatant **I** (see scheme 1 in the main text) after the real time in-cell ^{19}F NMR measurement at 4 °C. The integral intensity in supernatant is similar to that in control, i.e., all the trifluoroacetate ions remain outside cells. This is consistent with no detectable ^{19}F signal for the counterions in ^{19}F NMR spectrum of cell lysate fraction.

References

1. Jackson, J.C.; Hammill, J.T.; Mehl, R.A. Site-specific incorporation of a ^{19}F-amino acid into proteins as an NMR probe for characterizing protein structure and reactivity. *J. Am. Chem. Soc.* **2007**, *129*, 1160–1166.
2. Luchette, P.A.; Prosser, R.S.; Sanders, C. Oxygen as a paramagnetic probe of membrane protein structure by cysteine mutagenesis and ^{19}F NMR spectroscopy. *J. Am. Chem. Soc.* **2000**, *122*, 4408–4417.
